# Sago Starch Bionanocomposite Films With *Peganum harmala* and TiO_2_: Enhancing Oxidative Stability and Quality of Chicken Fillets

**DOI:** 10.1002/fsn3.71477

**Published:** 2026-01-20

**Authors:** Alireza Bagher Abiri, Homa Baghaei, Nurul Huda, Hendrix Yulis Setyawan, Abdorreza Mohammadi Nafchi

**Affiliations:** ^1^ Department of Food Science and Technology, Da.C. Islamic Azad University Damghan Iran; ^2^ Postgraduate School Universitas Brawijaya Malang East Java Indonesia; ^3^ Department of Agroindustrial Technology, Faculty of Agricultural Technology Universitas Brawijaya Malang East Java Indonesia; ^4^ Food Technology Division, School of Industrial Technology Universiti Sains Malaysia Penang Malaysia

**Keywords:** active film, meat, nano‐TiO_2_, oxidation, *Penganum harmala*, sago starch

## Abstract

In this study, we developed starch‐based bionanocomposite films by incorporating *Peganum harmala* extract (PE) and titanium dioxide (TiO_2_) nanoparticles into a sago‐starch matrix. We evaluated their physicochemical properties and their ability to retard lipid oxidation and preserve sensory quality in chicken fillets during 12 days of refrigerated storage (4°C). The optimal film formulation containing 3% TiO_2_ and 10% PE exhibited a significantly reduced moisture content (6.16% ± 0.37% vs. 10.53% ± 0.39% in the control), lower water solubility (18.74% ± 0.49% vs. 23.94% ± 0.82%), and enhanced water absorption capacity (1.60 ± 0.04 vs. 2.19 ± 0.07 g/g). Mechanical testing showed that its tensile strength increased by 54%, from 14.32 ± 0.59 MPa (control) to 22.01 ± 0.75 MPa, while elongation at break remained acceptable (20.60% ± 0.27%). When applied to chicken fillets, this active film reduced peroxide values by approximately 48% (from 9.2 to 4.8 meq O_2_/kg) and thiobarbituric acid–reactive substances by 42% (from 1.65 to 0.96 mg MDA/kg) after 12 days, compared to unwrapped samples. Sensory evaluation by a trained panel revealed that the odor, color, and overall acceptability scores of the coated fillets remained above 7.5 (on a 9‐point scale), whereas the control samples scored below 5. The improved oxidative stability is attributed to the synergistic effect of TiO_2_, a UV‐shielding agent, and PE, a radical scavenger. At the same time, the mechanical reinforcement is due to strong hydrogen bonding between the starch matrix and nanoparticles. These findings demonstrate that sago‐starch/TiO_2_/PE bionanocomposite films can effectively enhance oxidative stability and maintain sensory quality in refrigerated poultry, offering a promising biodegradable packaging solution.

## Graphical abstract

This study focuses on the development and characterization of active bionanocomposite films using a sago‐starch matrix reinforced with titanium dioxide (TiO_2_) nanoparticles and *Peganum harmala* extract. The results demonstrate that the incorporation of these additives significantly improves the mechanical and barrier properties of the films while effectively inhibiting lipid oxidation and maintaining the sensory quality of chicken fillets during 12 days of refrigerated storage. Overall, the research highlights the potential of these bionanocomposite films as a sustainable and functional packaging solution for extending the shelf life of high‐protein food products.

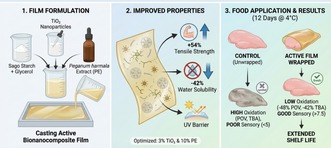

## 1 Introduction

Generally, fresh meats have a short shelf life when stored in the refrigerator and are considered perishable food products. During the cold storage period, they are easily spoiled by microbial growth and lipid oxidation (Choubaki et al. 2025). Packaging is recognized as the most crucial factor in protecting foods against environmental conditions, ensuring the safety and quality of packaged products (Dang, Han, and Wang 2025; Esfahani et al. 2022). In recent years, biopolymer packaging derived from natural sources, such as lipids, carbohydrates, and proteins, has been considered an effective solution to mitigate pollution caused by petroleum‐based packaging materials (Dang, Han, et al. 2025; Khajeh et al. 2025; Khoshdouni Farahani, Mahasti Shotorbani, et al. 2025). Sago starch is a renewable, biomass‐derived polysaccharide with excellent film‐forming ability, biodegradability, and wide availability, making it a promising alternative to petroleum‐based packaging materials (Ramli et al. 2025). However, these biopolymer packaging films often exhibit weak water barrier and mechanical properties, and they usually lack functional properties such as antioxidant and antimicrobial activity (Dang, Cai, and Wang 2025; Samani et al. 2023). Research has shown that biopolymer films can carry functional agents. Various bioactive additives, such as herbal extracts and essential oils, have been used in food packaging films in different studies to delay microbial growth and lipid oxidation in food products (Babaei et al. 2025; Hunashyal et al. 2025; Nikmanesh et al. 2023; Suthan et al. 2025). In this way, active packaging is developed, the purpose of which is to guarantee the safety and quality of food products, as well as improve their shelf life and maintain their appearance (Fang et al. 2017). *Peganum harmala* , also known as Espand, belongs to the Zygophylaceae family and is used in traditional medicine. It is used to treat respiratory diseases, gastrointestinal, endocrine, nervous, cardiovascular, skin and hair, pain relief, inflammation, neoplasms and tumors, antipyretic, diabetes, disinfectant, arthritis, ulcers, and rheumatism (Shahrajabian et al. 2021). This plant is native to the Mediterranean region, with its origin in Central Asia (Allaq et al. 2021). Various bioactive compounds have been detected in Espand extracts, whose functional activities, such as anti‐fungal, antibacterial, and anti‐viral, have been confirmed by previous researchers (Bagher Abiri et al. 2023). The noticeable antioxidant activity of Espand extract has also been proven (Abolhasani et al. 2015; Saeedeh et al. 2022). Anthroquinons, flavonoid glycosides, β‐carbolinalkaloids, and alkaloids are known as major bioactive compounds in Espand extract (Mohsenipour and Hassanshahian 2016).

Another widely used method to improve the functional properties, as well as the physical, mechanical, and barrier characteristics of biopolymer films, is the application of nanotechnology and inorganic nano‐fillers (Dang et al. 2024; Gunaki et al. 2025; Pinto et al. 2025). Titanium dioxide (TiO_2_), a three‐dimensional, non‐toxic, and low‐cost nanoparticle, has been used as an inorganic nanofiller in the food packaging industry over the past few decades. These nanoparticles have remarkable antimicrobial activity against a wide range of microorganisms (Dang et al. 2023; Tsai et al. 2010). The positive impact of these nanoparticles on the barrier, mechanical, physical, and thermal characteristics of packaging films based on natural biopolymers has been demonstrated by previous researchers (Sani et al. 2021; Sheibani et al. 2025; Zhang and Rhim 2022). Recent studies have shown the synergistic effect of organic nanoparticles and herbal extracts or essential oils (Javidi et al. 2022; Lan et al. 2021; Li et al. 2019).

While previous research has explored starch‐based films with various additives, this study is the first to examine the synergistic integration of TiO_2_ nanoparticles and *Peganum harmala* extract within a sago‐starch matrix. Unlike standard biodegradable films, this dual‐functional system specifically addresses the oxidative challenges posed by high‐protein muscle foods, providing a tailored solution for poultry preservation. The present study is the first to integrate *Peganum harmala* extract and TiO_2_ nanoparticles into a sago‐starch matrix to produce an active bionanocomposite film and to systematically evaluate their combined effects on the physicochemical properties of the packaging material and on the oxidative stability and sensory quality of chicken fillets under refrigerated storage conditions. By optimizing the concentrations of bioactive extract and nanofiller, we demonstrate a synergistic enhancement of barrier, mechanical, and antioxidant properties in the film, resulting in a significant reduction in lipid oxidation markers (peroxide value and TBARS) and the preservation of key sensory attributes over 12 days at 4°C. This dual‐functional biopolymer system not only advances the design of fully biodegradable, active packaging materials but also offers a scalable approach for extending the shelf life of perishable meat products without reliance on synthetic additives.

## 2 Materials and Methods

### 2.1 Materials

Sago starch (food grade) was procured from SIM Supply SDN Bhd (Penang, Malaysia), and glycerol (analytical grade, ≥ 99%) served as plasticizer (Merck, Darmstadt, Germany); titanium dioxide nanoparticles (anatase phase, ~20 nm, ≥ 99.5% purity) were obtained from Sigma‐Aldrich (St. Louis, MO, USA); *Peganum harmala* seeds were collected from wild populations in the Yazd region (Iran), authenticated by the University of Tehran's Department of Botany. DPPH, TBA, and other reagents for antioxidant assays (all Sigma‐Aldrich) and HPLC‐grade methanol (Fisher Scientific) were used as received; fresh chicken breast fillets (Pekin cross, 6–8 weeks old, 150–170 g) were procured from a local poultry slaughterhouse in Tehran, Iran, and transported on ice; deionized water (≥ 18 MΩ cm) was generated in‐house using a Millipore Milli‐Q system.

### 2.2 *Peganum harmala* Extract (PE) Preparation

*Peganum harmala* was collected, dried in the shade and at room temperature, and then ground into a powder. This powder (40 g) was then mixed with 100 mL of 80% ethanol for 24 h at room temperature on a magnetic stirrer (Heidolph, Germany). After that, the mixture was filtered (Whatman No. 1 paper) and the solvent was evaporated at 40°C using a rotary evaporator (Heidolph MR Hei‐standard, Germany).

### 2.3 Preparation of Sago Starch/Nano‐TiO_2_ /PE Films

Initially, solutions with different concentrations of Nano‐TiO2 (1%, 3%, and 5% w/w) were obtained by dissolving the appropriate amounts of Nano‐TiO2 in 100 mL of distilled water and homogenizing for 20 min in an ultrasonic bath (BANDELIN SONOREX digitec, Germany). The sago starch (4 g) and glycerol (2 g) were added to the Nano‐TiO_2_ solutions and stirred. The mixtures were heated to 90°C and then maintained at this temperature for 30 min to completely gelatinise the starch (Teymourpour et al. 2015). The temperature of the mixtures was then increased to 45°C, and different levels of PE (5%, 10%, and 15% v/v) were incorporated into the film solutions. After homogenizing the mixtures for 30 min, 50 g of the film solutions were poured into plates and dried overnight at room temperature. Finally, the films were conditioned in a desiccator (containing saturated Mg(NO_3_)_2_) (Bagher Abiri et al. 2023).

### 2.4 Film Characterization

#### 2.4.1 Moisture Content (MC), Water Solubility (WA), and Water Absorption Capacity (WAC) Measurement

The film pieces (3 × 3 cm) were conditioned in a desiccator at 55% RH overnight, and their weight was recorded (W0). After that, the films were dried overnight at 100°C in an oven and, upon cooling to room temperature, their weight was recorded (W_1_). Under stirring (100 rpm), the films were placed in distilled water (50 mL) for 60 min at room temperature. After removing their surface water with filter paper, the films were dried again in the oven, and their weight (W_2_) was obtained. The MC and WA values of the films were calculated using Equations (1) and (2). To determine the WAC of the films, after initially conditioning the samples in the desiccator, the films were placed in a container containing distilled water (50 mL) for 1 day under constant stirring; their weight was measured, and this process was repeated until the constant weight was reached. The final weight of the films was recorded (W_3_). The WAC values of the samples were calculated using Equation (3).

(1) $$\mathrm{MC}\;\left(\%\right)=\frac{W1-W0}{W0}\times 100$$

(2) $$\mathrm{WA}\;\left(\%\right)=\frac{W1-W2}{W2}\times 100$$

(3) $$\mathrm{WAC}\;\left(g\;\text{water}/g\;\text{dried film}\right)=\frac{W3-W0}{W0}$$

#### 2.4.2 Mechanical Properties Measurement

The mechanical properties of the films, including tensile strength (TS), Young's modulus (YM), and elongation at break (EAB), were measured using the ASTM D882 standard method and a texture analyzer device (LLOYD, RS 232, America). The films were conditioned overnight at room temperature and an RH of 57% in a desiccator (containing magnesium nitrate), and then the film piece (dimension of 8 × 0.5 cm) was placed between the grips of the device, and the mechanical test was performed at room temperature using a 50 kg cell bar. The initial distance between the grips was 50 mm, and the speed of the grips was 5 mm.min^−1^ (Shiryanpour et al. 2025).

#### 2.4.3 Color Measurement

The color of the films was studied using the Hunter Lab device (Minolta, America). The color indices investigated in this research included L* (brightness), a* (redness/greenness), and b* (yellowness/blueness) (Choubaki et al. 2025). Total color differences (ΔE) of the films were also obtained using Equation (4):

(4) $$\mathrm{\Delta E}=\sqrt{{\left(L0-\mathrm{Lt}\right)}^{2}+{\left(a0-\mathrm{at}\right)}^{2}+{\left(b0-\mathrm{bt}\right)}^{2}}$$

#### 2.4.4 UV–Vis Transmittance Measurement

The UV–Vis transmittance percentage of the films was investigated using a spectrophotometer (Shimadzu, model UV‐1700, Japan) at wavelengths ranging from 200 to 1100 nm (Hosseini et al. 2013).

### 2.5 Preparation of Chicken Fillets

The chicken breast was prepared fresh and chopped into 200 g pieces. The tests were conducted on fresh fillets and considered the data from the first day. The chicken fillets were then covered with the prepared films and stored in a refrigerator at 4°C for 12 days. The control sample had neither an edible nor an active film.

### 2.6 Chicken Meat Characterization During Preservation

#### 2.6.1 Peroxide Value (POV) Measurement

To measure the POV of chicken fillets, the oil from the samples was first extracted with chloroform‐methanol solvent, according to the method described by Soyer et al. (2010). Then, 5 g of the oil sample was mixed with a mixture of glacial acetic acid and chloroform (3:2) in a 250 mL Erlenmeyer flask. 0.5 mL of a saturated potassium iodide solution was added, and the mixture was kept in the dark for 5 min. After this time, 75 mL of distilled water and 0.5 mL of starch reagent were added, and the solution was then titrated with a 0.01 N sodium thiosulfate solution. In the blank test, all the mentioned steps were performed without the presence of the sample. Finally, the POV of samples was calculated using Equation (5) and reported in meq O_2_.kg^−1^ of oil (Javan et al. 2013). In this equation: *S* is the volume of thiosulfate used for the titration of the sample (mL), *B* is the volume of thiosulfate used for the titration of the blank (mL), *N* is the normality of sodium thiosulfate (eq mL^−1^), and *W* is the weight of the sample (g).

(5) $$\mathrm{POV}\;\left(\mathrm{meq}\;O_{2}/\mathrm{kg}\;\text{of oil}\right)=\frac{\left(S-B\right)\times N\times 1000}{W}$$

#### 2.6.2 Thiobarbituric Acid (TBA) Measurement

To measure the TBA value of fillet samples, 200 mg of the homogenized sample was weighed in a 25 mL flask, and the volume was made up with 1‐butanol. Then, 5 mL of the resulting mixture was transferred to a dried Falcon tube, and 5 mL of TBA reagent solution was added. The tube was heated at 95°C in a hot water bath for 2 h, cooled to ambient temperature, and then its absorbance (As) was read at 530 nm using a spectrophotometer against distilled water as the control (Ab). Finally, TBA values of samples were calculated using Equation (6) and expressed as mg MDA/kg of sample (Raeisi et al. 2014).

(6) $$\mathrm{TBA}\;\left(\mathrm{mg}\;\mathrm{MDA}/\mathrm{kg}\;\text{of sample}\right)=\frac{50\times \left(\mathrm{As}-\mathrm{Ab}\right)}{200}$$

#### 2.6.3 Sensory Evaluation

The sensory evaluation was conducted in accordance with the ethical principles for medical research involving human subjects outlined in the Declaration of Helsinki. Although a formal institutional ethics committee was not available, the study strictly adhered to safety and ethical protocols. All 10 trained panelists were fully informed of the study's objectives and the nature of the samples, and informed verbal consent was obtained from each participant before the sessions. To ensure participant safety, all film ingredients were of food‐grade quality, and the chicken fillets were prepared under hygienic conditions and cooked to a safe internal core temperature of 70°C to eliminate any microbiological risks. Color, odor, texture, and overall acceptability were scored on a 5‐point Hedonic scale, where 5 = “very good” and 1 = “very bad”, by the panelists (Ojagh et al. 2010).

### 2.7 Statistical Analysis

All experimental procedures, including the preparation of bio‐nanocomposite films and the treatment of chicken fillets, were conducted in three independent batches (*n* = 3) to ensure reproducibility. Each batch was analyzed separately, and the results are reported as the mean ± standard deviation. Statistical analysis was performed using SPSS software (version 22.0) and one‐way ANOVA (analysis of variance). The sample differences were analyzed at *p* < 0.05 using Duncan's multiple range test.

## 3 Results and Discussion

### 3.1 Moisture Content (MC), Water Solubility (WA), and Water Absorption Capacity (WAC) of Films

The barrier properties of packaging films against moisture are important characteristics of biopolymer films in maintaining the quality of food products, especially those sensitive to humidity (Zolfi et al. 2014). The Moisture barrier properties of sago starch films containing the combination of different levels of nano‐TiO_2_ and PE are given in Table 1. The MC, WA, and WAC values of the starch films in this research were in the range of 4.25%–10.53%, 15.84%–23.94%, and 1.25–2.19 g water/g dried film, respectively. The highest values of MC, WA, and WAC were observed in the control sample. Adding the combination of Nano‐TiO_2_ and PE to starch films resulted in a decrease in the values of these quality parameters compared to the control (*p* < 0.05). Increasing the level of Nano‐TiO_2_ from 1% to 5% in the films also caused a significant decrease in the MC, WA, and WAC values of the samples; however, at constant levels of nanoparticles, increasing the level of PE from 5% to 15% indicated a significant increase in these parameters (*p* < 0.05). Generally, the MC and WAC of the films depend on the space in the film structure and the hydrophilic characteristic of the film. By filling the film's voids and compacting the film structure, nanoparticles can reduce the MC and WAC of the films (Pirsa et al. 2020; Wu et al. 2009).

**TABLE 1:** Moisture content, water solubility, and WAC of sago starch/Nano‐TiO_2_/PE bionanocomposite films.

Film samples	MC (%)	WS (%)	WAC (g water/g dried weight)
Control	10.53 ± 0.39 a	23.94 ± 0.82 a	2.19 ± 0.07 a
1% TiO_2_ + 5% PE	8.78 ± 0.34 c	22.30 ± 0.69 b	1.78 ± 0.06 c
1% TiO_2_ + 10% PE	9.36 ± 0.23 b	23.16 ± 0.63 a,b	1.88 ± 0.04 c
1% TiO_2_ + 15% PE	9.92 ± 0.29 a	23.70 ± 0.58 a,b	1.99 ± 0.05 b
3% TiO_2_ + 5% PE	5.57 ± 0.31 e,f	18.01 ± 0.52 d,e	1.51 ± 0.07 e,f
3% TiO_2_ + 10% PE	6.16 ± 0.37 e	18.74 ± 0.49 c,d	1.60 ± 0.04 d,e
3% TiO_2_ + 15% PE	6.79 ± 0.18 d	19.22 ± 0.50 c	1.65 ± 0.03 d
5% TiO_2_ + 5% PE	4.25 ± 0.30 g	15.84 ± 0.77 g	1.25 ± 0.05 h
5% TiO_2_ + 10% PE	4.89 ± 0.36 g	16.89 ± 0.47 f,g	1.35 ± 0.03 g
5% TiO_2_ + 15% PE	5.55 ± 0.22 f	17.41 ± 0.55 e,f	1.47 ± 0.04 f

*Note:* Values represent mean (*n* = 3) ± SD. Different letters in each column indicate a significant difference at the 5% level of probability among films.

Abbreviations: MC, moisture content; WA, water solubility; WAC, water absorption capacity.

On the other hand, as a result of the reaction between nanoparticles and the film network and the reduction of free hydroxyl groups in the film network, the WA percentage of the samples also decreases, which is consistent with the results reported by other researchers (Din et al. 2021; Sani et al. 2019; Tamimi et al. 2021). The increase in the MC, WA, and WAC values of the films due to the rise in the level of PE is likely due to the presence of active compounds of a hydrophilic nature in this extract, such as polyphenols, which have enhanced the hydrophilic characteristic of the films. Similarly, previous studies have shown that the use of plant extracts increases the moisture sensitivity of biopolymer films (Ekramian et al. 2021; Mehdizadeh et al. 2020).

### 3.2 Mechanical Properties of Films

Mechanical characteristics are noticeable and important parameters that affect the integrity of the packaging film and also impact the packaging's resistance to external stresses. The mechanical properties of starch bionanocomposite films containing the combination of Nano‐TiO_2_ and PE, including tensile strength (TS), Young's modulus (YM), and elongation at break (EAB), are shown in Table 2. The values of TS, YM, and EAB of the films produced in this research were in the range of 14.32–28.73 MPa, 94.41–116.60 MPa, and 15.74%–26.46%, respectively. By adding a combination of Nano‐TiO_2_ and PE to the film samples, the values of TS and YM increased, and the EAB percentage of the films decreased significantly (*p* < 0.05). Increasing the levels of Nano‐TiO_2_ from 1% to 5% resulted in a significant increase in TS and YM and a decrease in the percentage of flexibility of the films (*p* < 0.05); however, at constant levels of Nano‐TiO_2_, with an increase in the level of PE from 5% to 15%, the TS and YM decreased and the EAB significantly increased (*p* < 0.05). In general, the formation of strong bonds between nanoparticles and the starch network in the film structure enhances the film's strength, resulting in a denser, stronger film (Mukurumbira et al. 2017). The decrease in the strength of the films and the increase in their flexibility, attributed to the increase in the PE level in the samples, is likely due to the plasticising role of plant extracts and their phenolic compounds. These results were consistent with the results observed by other researchers. For example, Vaezi et al. (2019), Hu et al. (2019), and Mirjalili and Yassini Ardekani (2017) also reported improvements in the mechanical strength of starch films due to the incorporation of nanoparticles. In the research by Mehdizadeh et al. (2020) and Ekramian et al. (2021), the addition of herbal extracts to biopolymer films decreased mechanical strength and increased flexibility.

**TABLE 2:** Mechanical properties of sago starch/nano‐TiO_2_/PE bionanocomposite films.

Film samples	TS (MPa)	YM (MPa)	EAB (%)
Control	14.32 ± 0.59 i	94.41 ± 1.33 g	26.46 ± 0.35 a
1% TiO_2_ + 5% PE	17.36 ± 0.61 g	102.77 ± 1.08 d	23.43 ± 0.30 d
1% TiO_2_ + 10% PE	16.00 ± 0.78 h	100.40 ± 0.96 e	24.52 ± 0.28 c
1% TiO_2_ + 15% PE	14.50 ± 0.53 i	98.12 ± 1.10 f	25.74 ± 0.33 b
3% TiO_2_ + 5% PE	23.62 ± 0.82 d	110.06 ± 1.05 b	19.38 ± 0.25 g
3% TiO_2_ + 10% PE	22.01 ± 0.75 e	107.82 ± 1.24 b	20.60 ± 0.27 f
3% TiO_2_ + 15% PE	20.43 ± 0.54 f	104.94 ± 0.99 c	21.49 ± 0.24 e
5% TiO_2_ + 5% PE	28.73 ± 0.88 a	116.60 ± 1.44 a	15.74 ± 0.26 j
5% TiO_2_ + 10% PE	27.34 ± 0.21 b	114.08 ± 1.13 a	16.67 ± 0.30 i
5% TiO_2_ + 15% PE	25.41 ± 0.64 c	110.99 ± 1.18 b	17.65 ± 0.24 h

*Note:* Values represent mean (*n* = 3) ± SD. Different letters in each column indicate a significant difference at the 5% level of probability among films.

Abbreviations: EAB, elongation at break; TS, tensile strength; YM, young modulus.

The improvement in tensile strength and reduction in moisture sensitivity observed in the present study are consistent with previous reports on starch‐based bionanocomposite films reinforced with inorganic nanoparticles and plant extracts (Choubaki et al. 2025; Dang, Han, et al. 2025).

### 3.3 Color of Films

In this research, the color indexes of starch‐based bionanocomposite films, including the brightness (L), redness/greenness (a), yellowness/blueness (b), and total color differences (ΔE), were determined according to the calorimetric method, and the results are presented in Table 3. The results showed that the control sample had the highest value of brightness (92.47) and the lowest values of redness (0.11) and yellowness (2.35), and adding the combination of Nano‐TiO_2_ and PE to the film samples and increasing the levels of these two additives in the films by reducing the L* Alirez and increasing the a* and b* indexes caused the color of produced films to become darker, redder, and yellower compared to the control (*p* < 0.05). With the increase in the levels of Nano‐TiO_2_ and PE in the films, the ΔE index showed a significant increase (*p* < 0.05). The lowest value of L* (57.51) and the highest values of a* (4.35), b* (21.23), and ΔE (39.96) were observed in the film containing the combination of 5% and 15% PE. One of the reasons for darkening the color of the starch films due to the addition of PE is the presence of phenolic compounds, as these active compounds can absorb light with low wavelengths and thus cause the color of the films to become darker (Shojaee‐Aliabadi et al. 2013). Another important reason is the color of PE, which contributes to the darker color of films containing this extract compared to the control film. The accumulation of nanoparticles in the structure of biopolymer films also reduces the color brightness of the films. So the films produced in this research can be used for packaging light‐sensitive foods. These results were in line with those of other researchers, who reported the darkening of the film's color due to the incorporation of plant extracts and nanoparticles (Mehdizadeh et al. 2020; Sani et al. 2019; Shiryanpour et al. 2025; Vaezi et al. 2019).

**TABLE 3:** Color properties of sago starch/Nano‐TiO_2_/PE bionanocomposite films.

Film samples	L*	a*	b*	ΔE
Control	92.47 ± 1.06 a	0.11 ± 0.04 i	2.35 ± 0.22 i	—
1% TiO_2_ + 5% PE	84.06 ± 0.87 b	1.56 ± 0.09 i	8.59 ± 0.31 i	10.57 ± 0.53 i
1% TiO_2_ + 10% PE	82.14 ± 0.81 c	1.76 ± 0.06 h	9.34 ± 0.28 h	12.56 ± 0.46 h
1% TiO_2_ + 15% PE	79.92 ± 0.95 d	1.87 ± 0.02 g	10.64 ± 0.25 g	15.14 ± 0.58 g
3% TiO_2_ + 5% PE	69.54 ± 1.20 e	2.81 ± 0.10 f	13.57 ± 0.20 f	25.67 ± 0.50 f
3% TiO_2_ + 10% PE	67.63 ± 0.57 f	3.05 ± 0.08 e	14.28 ± 0.27 e	27.71 ± 0.33 e
3% TiO_2_ + 15% PE	65.77 ± 0.64 g	3.24 ± 0.07 d	15.00 ± 0.23 d	29.71 ± 0.42 d
5% TiO_2_ + 5% PE	61.86 ± 0.76 h	3.82 ± 0.11 c	19.95 ± 0.16 c	35.50 ± 0.27 c
5% TiO_2_ + 10% PE	60.07 ± 0.69 i	4.08 ± 0.12 b	20.57 ± 0.17 b	37.38 ± 0.64 b
5% TiO_2_ + 15% PE	57.51 ± 1.03 j	4.35 ± 0.09 a	21.23 ± 0.24 a	39.96 ± 0.51 a

*Note:* Values represent mean (*n* = 3) ± SD. Different letters in each column indicate a significant difference at the 5% level of probability among films. L* (brightness), a* (redness/greenness), and b* (yellowness/blueness).

### 3.4 UV–Vis Transmittance Percentage of Films

**FIGURE 1:**
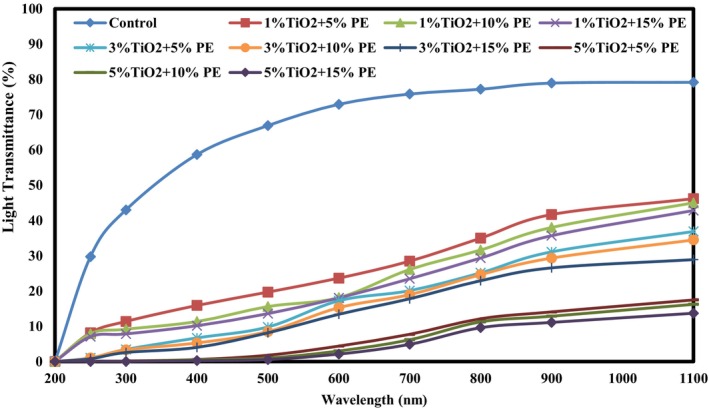
UV–Visible transmittance (%) of sago starch/Nano‐TiO_2_/PE bionanocomposite films.

In the food packaging industry, reducing the UV light transmission from the packaging films to the food inside is one of the main factors, because light can cause the development of lipid oxidation, leading to off‐odors and off‐colors in foods and reducing their nutritional value. The percentage of light transmittance in the visible and UV ranges of sago starch‐based bionanocomposite films is shown in Figure 1. As shown in the figure, the highest UV–Visible light transmittance was observed in the control film. Adding the combination of Nano‐TiO_2_ and PE to the films and increasing their levels led to a remarkable decrease in the percentage of UV–Visible light transmittance through the films. The effect of Nano‐TiO_2_ in reducing the percentage of UV light transmission was higher than that of PE. Previous research has reported results consistent with those of the present study, showing a notable decrease in UV–Visible light transmission through starch‐based films (Arezoo et al. 2020; Noorian et al. 2022; Rong et al. 2021). The effect of incorporating herbal extracts on reducing the percentage of UV–Visible light transmission from starch films has also been shown by other researchers (Homthawornchoo et al. 2022; Ju et al. 2019). Compared with similar biomass‐based packaging films derived from cassava, rice, or potato starch, the sago starch‐based films developed in this work exhibited physicochemical performance comparable to or superior to those of the other films, highlighting the suitability of sago starch as a sustainable packaging matrix (Hunashyal et al. 2025; Khoshdouni Farahani, Ebrahimzadeh Mousavi, and Mohammadi Nafchi 2025; Tayebi Rad et al. 2025).

### 3.5 Peroxide Value (POV) of Chicken Fillets

**FIGURE 2:**
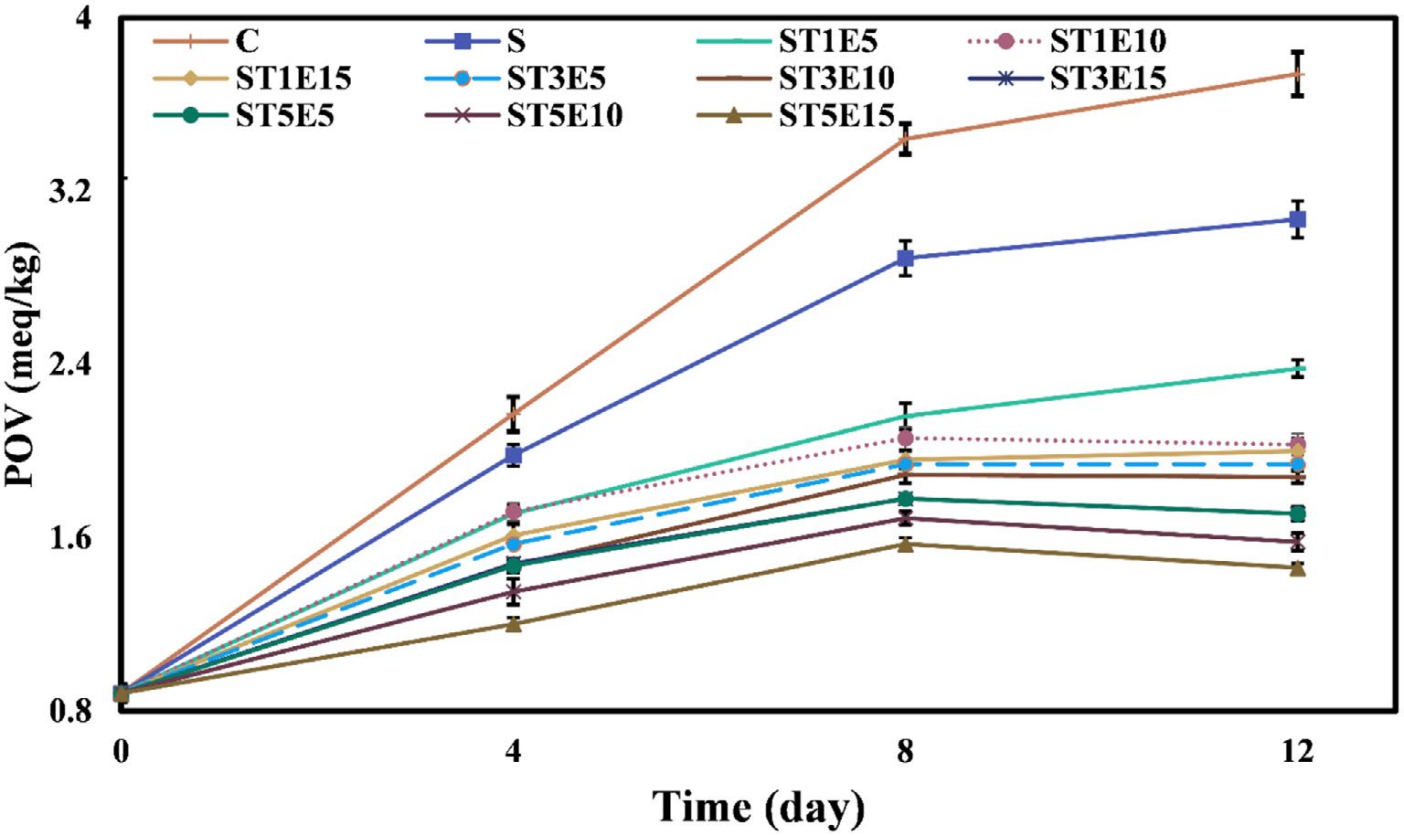
Changes in POV (meq O_2_/kg) of chicken fillets coated with sago starch/Nano‐TiO_2_/PE bionanocomposite films during a 12‐day storage period at 4°C. Error bars represent the standard deviation (*n* = 3). C, control; E, *Penganum harmala* extract; POV, peroxide value; S, samples coated with sago starch film without additives; T, nano‐TiO_2_.

Fats are susceptible to oxidation in the presence of various stimuli, including enzymes, heat, light, metals, microorganisms, and metalloproteins. During the process of lipid oxidation, hydroperoxides are continuously formed, which are the primary products of lipid oxidation. These hydroperoxides are subsequently broken down and converted into a wide range of volatile and non‐volatile secondary compounds. The POV test indicates the total amount of hydroperoxides (Shahidi and Zhong 2005). Changes in the POV of chicken fillets during the cold storage period are shown in Figure 2. Initially, the POV of fillets was 0.88 meq O_2_/kg. Over time, from the first day to the 8th day of cold storage, due to the development of lipid oxidation and the production of higher amounts of hydroperoxides, the POV of all samples increased significantly (*p* < 0.05). However, from the 8th to the 12th day, the POV increased in some samples, remained constant in others, and decreased in others (*p* < 0.05), reflecting differences in the rates of hydroperoxide formation and breakdown. At the end of cold storage, the highest POV was obtained in the control (3.74 meq O_2_/kg), followed by the fillets packed in starch film without additives (3.07 meq O_2_/kg), and the lowest value was for the sample packed in starch film containing the highest levels of Nano‐TiO_2_ and PE (5% Nano‐TiO_2_ and 15% PE) (1.46 meq O_2_/kg). These results indicate that the increase in the levels of Nano‐TiO_2_ and PE in the starch films enhances the antioxidant activity of the films, resulting in a decrease in the rate of lipid oxidation and fewer oxidative products formed in the meat samples. Some researchers have stated that the antioxidant activity of herbal extracts and essential oils is due to their reducing properties, which play a major role in neutralizing free radicals, deactivating singlet and triplet oxygen, and decomposing peroxides (Miguel 2010). The oxidation–reduction effect of inorganic nanoparticles is also related to reducing the transfer of oxygen and other gases from the environment surrounding the packaging to the food, as well as absorbing UV light. Similarly, in the research by Arfat et al. (2015), the POV of fish fillets initially increased and then decreased during cold storage. Still, the rate of change of this oxidative index in the fillets coated with active films containing ZnO nanoparticles and basil leaf essential oil was significantly less than that of the control sample. Previous researchers have stated that active bionanocomposite films act as an oxygen barrier, reducing the intensity of lipid oxidation by removing excess oxygen from the meat (Heydari‐Majd et al. 2019). Alizadeh‐Sani et al. (2020) also investigated the effect of active films based on whey protein/cellulose nanofiber containing TiO_2_ nanoparticles and rosemary essential oil on the quality of lamb meat and observed that the use of these active films can significantly decrease the rate of lipid oxidation and production of hydroperoxides in the samples compared to the control.

### 3.6 Thiobarbituric Acid (TBA) Index of Chicken Fillets

**FIGURE 3:**
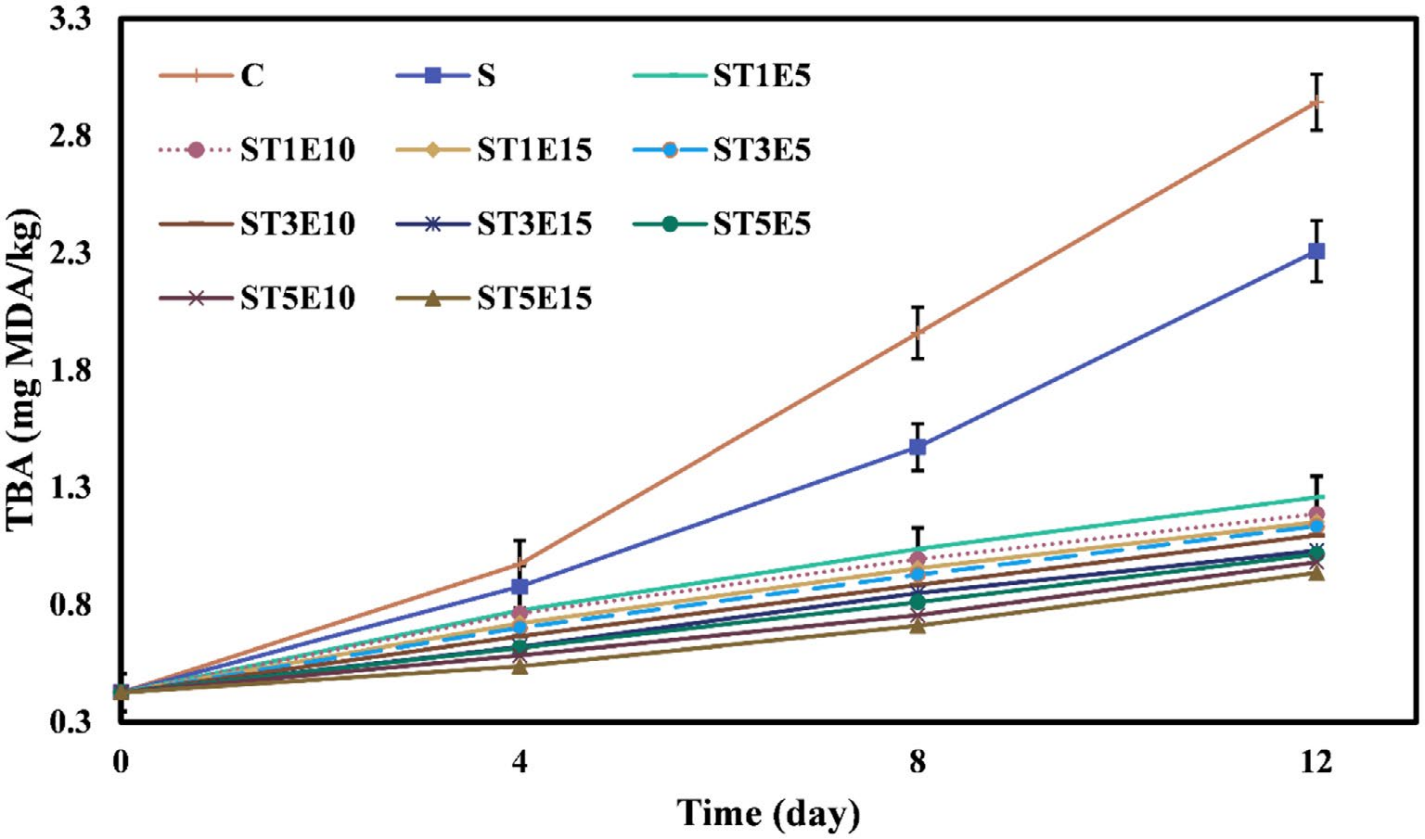
Changes in TBA (mg MDA/kg) of chicken fillets coated with sago starch/Nano‐TiO_2_/PE bionanocomposite films during a 12‐day storage period at 4°C. Error bars represent the standard deviation (*n* = 3). C, control; E, *Penganum harmala* extract; S, samples coated with sago starch film without additives; T, nano‐TiO_2_; TBA, thiobarbituric acid.

Hydroperoxides, primary products of lipid oxidation, are unstable and break down, producing secondary oxidation products such as ketones, aldehydes, and alcohols. TBA tests are used to measure malondialdehyde in food products. Changes in TBA values of chicken fillets during the cold storage period are shown in Figure 3. Initially, the TBA value of chicken fillets was 0.428 mg MDA/kg. During the cold storage period, due to the development of lipid oxidation and the further breakdown of hydroperoxides, as well as the formation of higher amounts of secondary oxidation products, the TBA values of all samples significantly increased (*p* < 0.05). The activity of psychrophilic bacteria during the cold storage period is also a contributing factor to the development of lipid oxidation over time, as these bacteria produce lipase and phospholipase enzymes, which lead to an increase in free fatty acids. As a result, they accelerate the rate of oxidation (Nirmal and Benjakul 2011). However, because the control sample lacked an active coating and natural antioxidants and antibacterial compounds, the highest malondialdehyde production was observed during the storage in this sample. As expected, at the end of the storage period, the highest values of TBA were for the control sample (2.942 mg MDA/kg) and the fillets packed in the film without additives (2.308 mg MDA/kg). The lowest value of TBA on the last day was related to the fillet packed in the starch films containing the highest level of Nano‐TiO_2_ in combination with the highest level of PE (5% Nano‐TiO_2_ and 15% PE) (0.935 mg MDA/kg). One of the reasons for the reduction of oxidative degradation in the chicken fillets with active films containing the combination of Nano‐TiO_2_ and PE is related to the role of these additives in reducing the oxygen permeability of the films. Because oxygen is one of the most important stimuli for lipid oxidation, and with the reduction of available oxygen, the rate of the oxidation process can also decrease.

On the other hand, reducing the UV transmission of UV light from the environment surrounding the packaging to the packaged meat can also lead to a reduction in the intensity of lipid oxidation in samples (Hajirostamloo and Molaveisi 2022). Additionally, PE contains various bioactive and functional compounds that exhibit remarkable antioxidant activity (Abolhasani et al. 2015). The effect of active biopolymer films containing herbal extracts and essential oils, as well as nanoparticles, in reducing the rate of lipid oxidation and producing smaller amounts of secondary products resulting from lipid oxidation in meats has been confirmed by other researchers (Alizadeh‐Sani et al. 2020; Azarifar et al. 2020; Mehdizadeh et al. 2020).

### 3.7 Sensory Evaluation of Chicken Fillets

**FIGURE 4:**
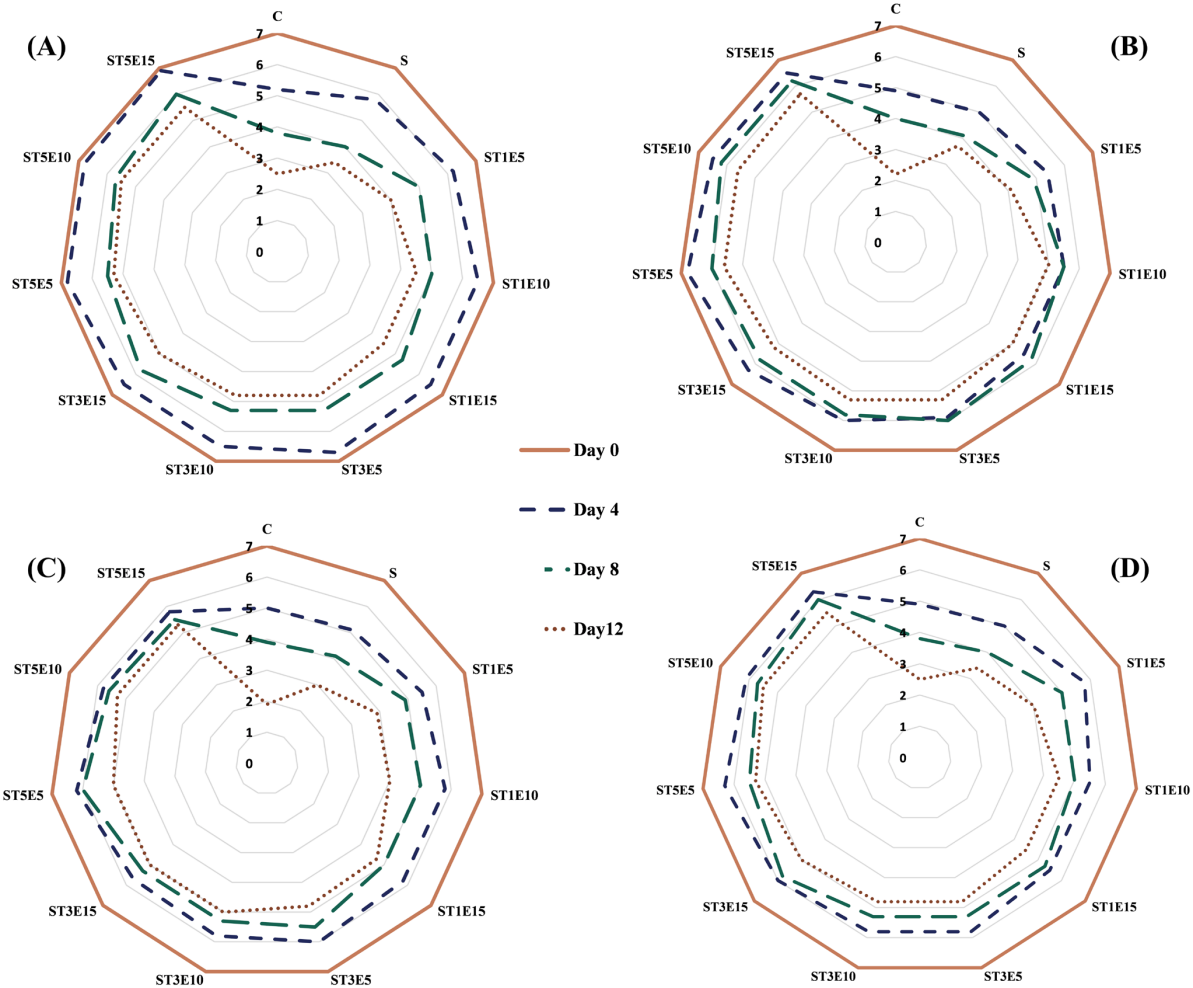
Comparison of the sensory scores (A: Texture, B: Color, C: Odor, and D: Overall acceptability) of chicken fillets coated with sago starch/Nano‐TiO_2_/PE bionanocomposite films during a 12‐day storage period at 4°C. C, control; E, *Penganum harmala* extract; S, samples coated with sago starch film without additives; T, nano‐TiO_2_.

The average scores of the sensory characteristics for color, texture, odor, and overall acceptability of chicken fillets during the cold storage period are compared in Figure 4A–D. At the beginning of the storage period, all the samples obtained full sensory scores. Due to the freshness of the chicken fillets used in this research, all the fillet samples were excellent in terms of various sensory properties. During the cold storage period, the scores of sensory properties of the fillets gradually decreased; however, the use of active films containing different combinations of Nano‐TiO_2_ and PE was able to maintain the sensory quality of the fillets better. In the samples packed with active films in high concentrations of PE on the 4th day of storage, there was a slight decrease in color and odor scores due to the migration of aromatic compounds present in this extract; however, these changes were generally accepted from a sensory point of view. The best sensory acceptance was achieved on the last day of cold storage, specifically for samples packed in starch films containing the highest levels of nanoparticles and extract. Generally, the decrease in odor scores of meat samples during storage is attributed to the breakdown of proteins by internal or microbial proteases, as well as the formation of volatile nitrogenous compounds (Raeisi et al. 2015). Lipid oxidation also affects the odor, color, and acceptability of fillets, thereby reducing their quality.

On the other hand, the growth of microorganisms also reduces the quality of the samples through the decomposition of the meat texture and the creation of an unpleasant smell. The decrease in the moisture content of the samples during storage can also be another reason for the reduction in the texture score during this period. The use of bionanocomposite films preserves the sensory quality of the meat fillets by reducing the growth rate of microorganisms and lipid oxidation, as well as preventing moisture loss during the storage period. Talebi et al. (2018) also reported that the use of an active film containing cellulose nanoparticles and peppermint essential oil was able to preserve the sensory quality of meat samples during cold storage. Alizadeh‐Sani et al. (2020) also reported that covering lamb meat samples with an active bionanocomposite film containing TiO_2_ nanoparticles and rosemary essential oil maintained the sensory quality of the samples during storage, a finding consistent with the results of the present study.

## 4 Conclusion

In this study, we successfully formulated a sago–starch–based bionanocomposite film incorporating 10% *Peganum harmala* extract and 3% TiO_2_ nanoparticles, which exhibit superior barrier and mechanical properties relative to the plain starch matrix. The optimized film demonstrated a 42% reduction in water solubility, a 54% increase in tensile strength, and maintained an elongation at break above 20%, while also delivering significant antioxidant activity (DPPH scavenging > 65%). When applied to chicken breast fillets stored at 4°C for 12 days, the active film decreased peroxide values by 48% and TBARS by 42% compared to unwrapped controls, and sensory scores for odor, color, and overall acceptability remained above 7.5 on a 9‐point hedonic scale. These results confirm that the dual incorporation of *P. harmala* extract and TiO_2_ nanoparticles produces a synergistic effect, effectively mitigating lipid oxidation and preserving sensory quality in refrigerated poultry. From a sustainability perspective, the use of sago starch as a biomass‐based polymer matrix, combined with plant‐derived *Peganum harmala* extract and low‐toxicity TiO_2_ nanoparticles, aligns with current efforts to develop eco‐friendly and biodegradable food packaging materials. Compared to conventional synthetic plastics, the developed film offers reduced environmental impact while maintaining functional performance suitable for meat preservation applications. Given its biodegradability and scalability, this bionanocomposite film represents a promising alternative to conventional synthetic packaging for extending the shelf life of perishable meat products.

## Author Contributions

**Nurul Huda:** conceptualization, writing – review and editing, funding acquisition, visualization. **Hendrix Yulis Setyawan:** writing – review and editing, validation, supervision, software. **Abdorreza Mohammadi Nafchi:** methodology, formal analysis, project administration. **Alireza Bagher Abiri:** investigation, writing – original draft preparation, validation. **Homa Baghaei:** supervision, data curation.

## Conflicts of Interest

The authors declare no conflicts of interest.

## Data Availability

The data that support the findings of this study are available from the corresponding author upon reasonable request.
